# Putting Pork on the Menu: Consumers’ Acceptability of Pork Meals with and Without Naturally Wood-Smoked Bacon

**DOI:** 10.3390/foods15193442

**Published:** 2026-09-26

**Authors:** Annchen Mielmann

**Affiliations:** The Africa Unit for Transdisciplinary Health Research (AUTHeR), Faculty of Health Sciences, North-West University, Potchefstroom 2531, South Africa; annchen.mielmann@nwu.ac.za

**Keywords:** meat, flavour, taste, sensory acceptability, emotions

## Abstract

This study aimed to determine consumers’ sensory acceptability of pork meals with and without added, naturally wood-smoked bacon to understand its value in driving local pork choices. Data for the study was collected in two phases. For phase I, respondents (*n* = 76) sensory evaluated six naturally wood-smoked bacon samples. For phase II, respondents (*n* = 63) sensory evaluated three pork meals without bacon and three pork meals with bacon. In phase I, rindless streaky bacon was the most liked as respondents moderately to very much liked it for its appearance, aroma, flavour and texture. The key sensory attributes for the streaky bacon were a golden colour with more meat than fat, a meaty and light smoky aroma, a salty and meaty flavour, and a juicy yet crispy texture. Across all six bacon cuts, respondents identified “happy” as the primary evoked emotion. In phase II, schnitzel with and without streaky bacon were selected as the most preferred pork meals. For the pork meals with bacon, the appearance, aroma, flavour and texture of the burger and both the rib burger and schnitzel differed significantly (*p* < 0.001). The emotions “happy”, “calm” and “interested” increased when bacon was added to the rib burger and schnitzel meals. Cohen’s d effect sizes were computed to determine the practical significance of demographic variations in pork meal acceptance Significant (*p* < 0.001) associations between the demographics, gender, age, education and income, and the acceptance of pork meals with and without bacon, were revealed. The concept of using naturally wood-smoked bacon could result in a positive engagement with pork and change consumers’ perception and perceived value of pork when choosing menu items.

## 1. Introduction

Globally, pork constitutes approximately 30% to 34% of both aggregate meat consumption and production, positioning it as a cornerstone of the international livestock sector [1]. Global pork consumption has grown significantly alongside rising populations, while South Africa has seen domestic pork consumption surge by 38.5% (nearly 90 thousand tons) between 2015 and 2024, outpacing marginal increases in beef and poultry and a decline in sheep meat contributing over 139 thousand tons and R6.7 billion to the country’s manufactured meat sector [2,3].

Pork meat cuts that are “naturally wood-smoked” refer to a process by which the meat is allowed to absorb smoke from smouldering fires using real hardwood chips [4]. This gives added flavour and colour to meat without using artificial smoke flavouring. Naturally wood-smoked pork products usually include bacon (cuts, diced, minced, rindless back, rindless streaky, round cut, shoulder), smoked ham, pulled pork, wors and ribs [5].

The smoking process is achieved by thermal combustion or by the direct application of liquid smoke into foods. The meat is exposed to the smoke of burning wood chips and pyrolysed to temperatures of 500 °C and above, resulting in the release of aromatic volatile compounds. The main aroma compounds found in bacon are aldehydes, pyrazines, pyridines, alcohols, ketones and furans. The application of the liquid smoke method eliminates carcinogenic compounds (e.g., polycyclic aromatic hydrocarbons) and allows higher process control, contributing to the desired aroma and flavour of conventional smoking. Due to the variety of naturally wood-smoked pork cuts available on the market, this research project will focus on bacon [6].

Bacon, a smoked and cured product, is one of the most eaten pork meat products in the world. Smoked bacon is consumed for its sensory attributes such as its distinctive colour, texture and satisfactory flavour [6,7]. Sensory characteristics such as colour, appearance, flavour and taste are important aspects to determine sensory quality of meat [8]. The processing steps of bacon involve trimming and skimming, curing, rinsing, soaking, slicing and packaging. Chemical reactions such as lipid oxidation and Maillard reactions generate volatile compounds that could contribute to bacon’s unique flavour [9]. A recent study found that ketone 3 methyl-1,2-cyclopentanedione in bacon may serve as a flavour enhancer with a prominent sweet and caramelized aroma [6]. Besides that, bacon is known for its savoury flavour umami—the fat or lipids present enhance the flavour developed in the smoking process [10].

The growth of the South African bacon segment reflects deeper macroeconomic and cultural shifts, wherein rapid urbanization and the demand for convenience dietary options heavily influence processed meat consumption patterns. South Africa’s bacon market is projected to generate USD 2.592.2 million in revenue by 2030, driven by an expected compound annual growth rate (CAGR) of 3.2% from 2024 to 2030 [11].

While academic consensus suggests that economic variables are not the primary drivers of pork consumption in South Africa, the cost–benefit implications of integrating pork into local diets warrant rigorous consideration, particularly against the backdrop of persistent domestic food insecurity. Despite the expansion of the South African middle class, a substantial segment of the population remains vulnerable to severe food poverty. Empirical data indicates that 13.8 million South Africans experience food poverty [12], while approximately 25% of the population reported depleting their financial resources to purchase food within a single calendar year [13].

In South Africa, pork meat continues to fall behind the demand for beef and chicken due to local myths and religious beliefs associated with the meat; however, of all meat types, pork production has marginally started to increase in South Africa due to its affordability, versatility and high protein content. Recent research revealed that consumers’ willingness to consume pork meat largely depends on how pork products are being prepared/cooked and presented [14]. As the consumption of pork meat is gradually gaining momentum [15], there is currently a lack of sufficient and sensory acceptable pork meal items on restaurant and fast-food menus for consumers to choose from.

Consumers’ emotions vary according to whether they like or dislike the food product [16]. Generating positive emotions enhances the overall eating experience, driving enjoyment and the motivation to consume meat. Specifically, when the dining experience evokes satisfaction, a consumer’s liking for the product rises. While certain emotions are universally felt across most meats, others are product-specific [16,17]. The fundamental contrasting emotions in this process are pleasure and its counterpart, displeasure or disgust. Researchers investigated the effect of hickory-smoked beef, bacon and basil aromas on consumers’ perceptions of food quality, emotions and the restaurant environment. The bacon aroma was found to notably enhance emotion, food quality perception, pleasure and overall perception of food and restaurant quality [18]. Bacon is also considered as a natural mood enhancer that helps to evoke a positive mental state. Despite the important role of emotions on consumers’ food choice and product market success, research on consumers’ emotions of meat and meat products are limited [19]. Consumers often experience pleasant, surprise or admiration when consuming palatable, high-quality meat [20]. However, adverse sensory qualities can elicit disgust, acting as a critical barrier to overall acceptance. This can be a result of some consumers are more sensitive to the distinctive odour or taste from boar taint in pork meat products. Boar meat acceptability is largely influenced by the demographics of the study participants and their baseline frequency of pork consumption [21]. Furthermore, the severity of boar taint and its subsequent detection by consumers can be modulated by the cooking procedure, meat type, production methods, and the age of the animal—especially in intact males prior to the onset of puberty [22].

As the local perception of pork gradually improves, it is gaining popularity among consumers. However, to grow this market segment optimally, consumer preferences must be better understood. Investigating the addition of naturally wood-smoked bacon to pork meals could drive pork choices in South Africa, nudging consumers toward a wider range of sensorily acceptable pork products. Therefore, the aim of this study is to determine consumers’ sensory acceptability of pork meals with and without added, naturally wood-smoked bacon to understand its value in driving local pork choices.

## 2. Materials and Methods

### 2.1. Ethical Approval

This study obtained ethical approval (NWU-00052-25-A1) from the Health Research Ethics Committee (HREC) of the Faculty of Health Sciences, North-West University, South Africa.

### 2.2. Sample and Data Collection

The target sample for this study was adult pork meat-eaters with access to social media. Vegan and vegetarian consumers were excluded from this study, as respondents needed to be familiar with pork consumption to gauge the emotions and perceptions of meat-eating consumers. This guaranteed that the data acquired through the online questionnaire did not include the opinions of plant-based consumers, minimizing potential bias. Respondents who showed any allergies, sensitivities, or intolerances to any ingredients in pork products were also excluded.

The study implemented a non-probability convenience sampling method. For recruitment, an electronic advertisement was posted via social media platforms (i.e., Facebook and LinkedIn) to reach the pork meat-eaters. Respondents scanned a QR code which redirected them to an online screening questionnaire to register for the study.

Data for the study was collected in two phases. In the first phase (April 2025), respondents (*n* = 76) sensory evaluated six naturally wood-smoked bacon samples and evaluated it in terms of their sensory acceptability and descriptives to determine the naturally wood-smoked bacon sample with the highest acceptance to use in the second phase of the study. In the second phase (May 2025), respondents (*n* = 63) sensory evaluated six prepared pork meal samples of which three meal samples were served without any bacon cuts and three pork meal samples were presented with the most accepted naturally wood-smoked bacon cut from phase I.

### 2.3. Pork Meat Samples and Meal Preparation

All pork meat cuts were purchased from a reputable retailer in South Africa and stored in a cold room (±2 °C). The naturally wood-smoked bacon included six different cuts (shoulder, rindless streaky, round cut, diced, rindless back and bacon cuts). For the pork meals, three types of pork products (schnitzel, burger and rib burger) were prepared. Commercially available convenience pork meals from the South African retail space were chosen because they are familiar to consumers and suitable for restaurant and fast-food menus. Driven by consumer demand for convenience and indulgence, a distinct market preference exists in South Africa for premium, high-value-added processed meat products like ribs and bacon [23].

On the day of each sensory evaluation session, all pork products were hygienically removed from the packaging and immediately cooked. All bacon cuts were pan fried over medium heat for 12 to 15 min, turning regularly until it was cooked and reached the desired crispiness. The crumbed pork schnitzels were oven baked in a pre-heated oven at 180 °C for 20 min until cooked. The burger patties were grilled at 180 °C for 8 min, turned and grilled for another 8 min until cooked to preference. The rib burger patties were oven baked at a pre-heated oven at 200 °C for 12 min, turned frequently and cooked to preference. All raw pork products were cooked to an internal temperature of at least 63 °C before removing meat from the heat source [24].

To prevent the range of temperature called “danger zone” (5–57 °C) [25], before serving the cooked pork meat products to the respondents, all pork meat products were kept warm on a warming tray above 60 °C for less than 30 min.

### 2.4. Sensory Evaluation of Pork Meat Samples

Sensory evaluation took place at the Consumer Sciences’ Sensory Laboratory at the North-West University, Potchefstroom, South Africa. The evaluation area of the Sensory Laboratory is a neutral room consisting of twenty individual booths, free of any odour and noise, with controlled temperature. For the bacon samples, each respondent received one sample tray of six naturally smoked-wood bacon cuts (Phase I).

For the pork meals (Phase II), each respondent received two sample trays of which the first tray contained three pork products (schnitzel, burger and rib burger) without bacon as to prevent sensory fatigue and severe carry-over effects, and the second tray contained the same pork products but with bacon. All samples were labelled with random three-digit codes and respondents received filtered water for palette cleansing. Respondents were requested to read the basic instructions before tasting the pork samples.

### 2.5. Questionnaire

For both phases, an online questionnaire was administered using Google Forms version 9.1.5.6. Each respondent received a tablet to access the online questionnaire.

For the first phase of the study, respondents scored the degree of liking of the six bacon cut samples using a nine-point hedonic scale (1 = Like extremely to 9 = Dislike extremely) in the following order: appearance, aroma, flavour and texture [4].

The “check-all-that-apply” (CATA) method is a practical method for collecting hedonic responses [26]. Respondents are provided with a list of descriptors and instructed to check all that apply to each sample. This approach enables the estimation of perceived attribute intensity based on the selection frequency among consumers. The CATA method is a reliable measure for the sensory characterization of consumer-related products as it is considered as a more consumer-friendly and faster approach to use when compared to trained panellists that require more time and is more resource intensive [26,27]. The CATA method allows the segmentation of consumers based on their preferences as a sensory profile is created for the food product [28]. Researchers determined the hedonic and descriptive sensory perception of bacon produced using alternative smoking processes and used the CATA method to assess consumers’ overall liking of the bacon products [6].

To determine the sensory descriptives for the bacon cut samples, the respondents completed CATA questions consisting of 38 descriptive attributes [4] that followed the sequence of evaluation in terms of sensory modality: (1) appearance (9 descriptives); (2) aroma (10 descriptives); (3) flavour (10 descriptives) and (4) texture (9 descriptives). To determine consumers’ food evoked emotions, each respondent indicated by means of the CATA method how strong they feel about each of the bacon cuts while tasting the bacon samples [29]. For the second phase of the study, respondents used the same measures to determine the acceptance and food-evoked emotions for the three pork meal samples, with and without bacon. Socio-demographic information such as age, gender, level of education and income status were also requested.

### 2.6. Data Analysis

Statistical analyses were performed with IBM SPSS Statistics (Statistical Package for Social Science, ver. 25.0, Chicago, IL, USA). Means and standard deviations were reported for sensory acceptance items measured on a Likert scale. Independent *t*-tests [30] and one-way ANOVAs [31] were performed for two- and three or more independent groups, respectively.

To detect practical significant differences between standardized means, Cohen’s d will be calculated [32]. A frequency analysis was performed to calculate the frequency of consumers’ answers to the CATA questionnaire. All statistical outputs were set at *p* < 0.05 for all analysis.

## 3. Results

### 3.1. Sensory Acceptance of Naturally Wood-Smoked Bacon

For phase I, rindless streaky bacon was the most liked as respondents moderately to very much liked it for its appearance, aroma, flavour and texture (Table 1). For appearance, rindless streaky bacon was significantly (*p* < 0.001) higher than round cut and diced bacon. The aroma for rindless streaky bacon was significantly (*p* < 0.001) higher than shoulder, round cut and bacon cuts. For flavour, rindless streaky bacon was significantly (*p* < 0.001) higher for shoulder and round cut bacon. The texture for rindless streaky was significantly (*p* < 0.001) higher than round cut, shoulder and diced bacon.

### 3.2. Sensory Attributes of Streaky Bacon

The most important sensory attributes for the streaky bacon were a golden colour and more meat than fat appearance, a meaty and light smoky aroma, a meaty and salty flavour, and a juicy and crispy texture (Figure 1).

### 3.3. Bacon-Evoked Emotions

Respondents indicated “happy” to be the most evoked emotion for all six bacon cuts. Interestingly, respondents revealed “calm” was the least evoked emotion and “interested” the most evoked emotion for rindless streaky bacon (Figure 2). A previous study found bacon aroma to notably enhance emotion, food quality perception, pleasure and overall perception of food and restaurant quality [18].

### 3.4. Sensory Acceptance of Pork Meals

Results from phase II of the study revealed that both the schnitzel with and without bacon were selected as the most preferred pork meals (Table 2). For the burger with bacon, the appearance and aroma improved. For the rib burger with bacon, the appearance and flavour were enhanced. For the schnitzel with bacon, appearance, aroma and flavour improved.

Regarding the sensory properties of the pork meals, for those meals without bacon, the appearance of the burger and schnitzel was significantly (*p* < 0.001) different. The aroma, flavour and texture of the burger and both for the rib burger and schnitzel were significantly (*p* < 0.001) different. For the pork meals with bacon, the appearance, aroma, flavour and texture of the burger and both the rib burger and schnitzel differed significantly (*p* < 0.001).

### 3.5. Pork Meal-Evoked Emotions

Respondents indicated “happy” to be the most evoked emotion for both the rib burger- and schnitzel meals with and without bacon. Interestingly, the emotions “sad”, “scared” and “disgusted” were associated with the burger meals (Figure 3).

Consumers were interested in the rib burger and schnitzel meals with bacon. The emotions “happy”, “calm” and “interested” increased when bacon was added to the rib burger and schnitzel meals with bacon.

### 3.6. Associations Between Demographics and Sensory Acceptance of Pork Meals

In Table 3, significant (*p* < 0.001) associations between demographics and the acceptance of pork meals without and with bacon are shown. For gender, male respondents indicated associations between appearance and the burger meals, and aroma and the rib burger meals. Both appearance and aroma increased for pork meals with bacon. Female respondents revealed relationships between both flavour and texture for the burger meals. Interestingly, both flavour and texture increased for the burger meals without bacon.

For age, the younger (18 to 44 years) respondents associate appearance, flavour and texture with the burger meals. For burger meals with bacon, the appearance increased, however for burger meals without bacon, the flavour and texture increased. Interestingly, the older (45 to 65+ years) respondents only associate appearance with the rib burger meals. For rib burger meals without bacon, the appearance elevated.

For highest level of education, only the respondents with an education level of Grade 12 or below indicated significant (*p* < 0.001) associations for appearance with the schnitzel and burger meals, aroma for the burger- and rib burger meals, and flavour for the burger meals. The appearance of schnitzel with bacon increased. The appearance and aroma for burger meals with bacon elevated, and the flavour for burger meals without bacon increased.

For income, respondents with a lower monthly income showed relationships for appearance, aroma, flavour and texture for the burger meals, and aroma for the rib burger meals. The appearance of burgers with bacon increased. The aroma for rib burger and burger meals with bacon also elevated. The flavour and texture of burgers without bacon increased. For respondents earning a higher income (R27,001–R100,000+), the aroma for burgers with bacon elevated.

## 4. Discussion

### 4.1. Sensory Acceptance of Bacon

For phase I, rindless streaky bacon emerged as the most preferred sample, with respondents rating it moderately to very much liked for appearance, aroma, flavour, and texture (Table 1). This high consumer acceptability aligns with existing literature indicating that the elevated lipid profile of belly-cut (streaky) pork enhances overall palatability and sensory drivers compared to leaner anatomical cuts [33]. The appearance scores for rindless streaky bacon were significantly higher (*p* < 0.001) than those for the round cut and diced bacon variations. Visually, the lean muscle and intermuscular fat characteristic of belly cuts create a highly attractive aesthetic profile that directly drives consumer purchasing intent [34]. Rindless streaky bacon exhibited significantly greater aroma scores (*p* < 0.001) than shoulder, round cut, and general bacon cuts. This finding is supported by research on volatile compounds, which establishes that thermal lipid degradation and fatty acid oxidation during cooking generate key volatile aromatic compounds such as hexanal and specific furans that are noticeable in high-fat pork belly matrices [35].

Regarding flavour attributes, rindless streaky bacon scored significantly higher (*p* < 0.001) than both the shoulder and round cut variations. Lipids operate as primary flavour precursors and hydrophobic carriers in matrix systems. The elevated fat-to-lean ratio characteristic of the streaky belly primal generates a more pronounced, complex savoury and umami profile upon thermal processing. In contrast, leaner cuts like the loin (round cut) or the complex muscle structures of the shoulder do not have enough fat to sustain rich flavour development and long-lasting taste [36,37,38]. The texture scores for rindless streaky bacon were significantly higher (*p* < 0.001) than those for round cut, shoulder, and diced bacon. Mechanical evaluations confirm that cooking renders the intramuscular lipids of pork belly, driving a distinct structural transition that balances crispness and tenderness. Conversely, cuts with lower fat content, such as the round or loin, experience greater moisture loss [39,40].

### 4.2. Sensory Profile of Streaky Bacon

Streaky bacon, a cut from the pork belly, consists of distinctive layers of meat and fat, providing a richer flavour and excellent mouthfeel. During the cooking process, the fat renders down, creating a moist centre and crisp edges. Appearance such as the meat’s colour is widely regarded as a significant decision factor when consumers purchase meat products [41,42]. As pork meat temperature increased, redness became more pronounced, enhancing the golden-brown colour preferred by consumers [43]. Temperature drives Maillard reactions, producing appealing flavour compounds [44]. Researchers analysed the aroma and flavour profiles of commercial Chinese traditional bacon across various geographical regions and characterized both meaty aroma and flavour attributes [45].

A salty taste plays an important role in serving as a potential mood and taste enhancer in food products [46]. A study by [47] confirmed the salt content influenced the flavour of bacon. Researchers evaluated how Brazilian consumers sensory perceive smoked bacon and confirmed the drivers of liking for consumers were the attributes related to smoky aroma, juiciness and texture. Furthermore, during consumption, the aroma and texture descriptives of bacon are the primary drivers of liking and affect even the noticeable sensory characteristics of the product [6]. Depending on consumer preferences, these attributes can indicate either positive or negative meat characteristics [10].

### 4.3. Emotional Responses to Bacon

Across all six bacon cuts, respondents consistently reported “happy” as the primary evoked emotion (Figure 2). Literature reveals that high-fat, savoury food matrices, such as bacon, elicit positive, high-hedonic and elevated-valence emotional responses. These affective profiles are mediated by physiological reward mechanisms that associate energy-dense substrates with adaptive survival behaviours [48]. Meat products with complex, pleasant aromas, such as the smoke and savoury notes characteristic of cured pork, usually trigger high positive emotional valence.

Volatile organic compounds generated during natural wood smoking (e.g., phenols, guaiacol) are strongly linked to high consumer acceptance [48,49]. Bacon is culturally recognized as a comfort food associated with indulgence, culinary pleasure and positive breakfast or social memories. A study on meat product sensory consumer research confirmed positive feelings like “happy” and “satisfied” serve as strong, direct predictors of overall consumer liking and future purchase intent [50].

The finding that rindless streaky bacon primarily stimulated interest while minimizing calmness triggers a physical and emotional response uniquely suited to this product. In Russell’s Circumplex Model of Affect, emotions are mapped across valence (pleasant vs. unpleasant) and arousal (activation vs. deactivation). Both “happy” and “interested” are high-arousal, positive emotions whereas “calm” is a low-arousal state [51]. The sensory profile of rindless streaky bacon appears to cause high activation rather than relaxation. A study on consumers’ dynamic sensory perception of bacon indicates that distinct consumer segments are highly sensitive to fat-to-lean ratios and texture profiles. The absence of the rind enhances both visual appeal and expected texture, rendering the product more accessible and potentially crisper. According to consumer behaviour research on novel and traditional food textures, foods that present complex or shifting oral textures (juiciness, crispiness, melting fat) stimulate high cognitive engagement [52]. This prevents a state of passive calmness and instead induces active focus or interest as the brain decodes the intense sensory stimuli during mastication [53].

### 4.4. Sensory Appeal of Pork Meals

This study found that adding bacon improved the aroma of both the burger and schnitzel, while also enhancing their overall flavour profiles. This improvement can be attributed to the complex chemical matrix of cured and smoked meats. Bacon fat has a high content of unsaturated fatty acids that undergo lipid oxidation during cooking, yielding volatile aldehydes (e.g., hexanal and heptanal) and ketones that drive a rich, savoury aroma that increases consumer overall liking [54].

The appearance of all three pork meals improved with the addition of bacon. Studies evaluating bacon’s sensory properties show that the inclusion of these stabilized red and pink tones provides a strong visual contrast against regular pork, preventing the unappealing greyish tint that cooked fresh pork can sometimes develop. This enhances the overall visual appeal, thereby promoting consumer acceptance [55].

The study noted that even without bacon, the appearance of the burger and schnitzel differed significantly (*p* < 0.001), while aroma, flavour and texture varied across all pork meals (with or without bacon). Comparative studies show that the way bacon fat melts depends entirely on the structure of the meat as it behaves differently when layered versus when mixed into a ground matrix. For instance, in a structured item like a rib burger, the fat release coats the palate differently than it does in a breaded schnitzel, resulting in varied sensory profiles [56].

### 4.5. Emotional Responses of Pork Meals

The introduction of bacon to both the rib burger and schnitzel meals led to increases in “happy”, “calm” and “interested” responses. In the literature, conventional meat products are linked to positive hedonic experiences, comfort and satisfaction. Consumers generally associate familiar, well-liked meat preparations with pleasure and well-being, which explains why “happy” serves as the baseline food-evoked emotion across both types of pork meals [57].

Bacon acts as a sensory enhancer due to its high fat content, saltiness, and distinct smoky aroma. According to research on meat palatability drivers, complex flavours and umami characteristics heavily dictate overall liking and positive emotional profiles. Furthermore, bacon is a highly recognizable and its presence elevates consumer interest via visual and aromatic cues and promotes a sense of satiety and calm through fatty-acid reward mechanisms in the brain [22].

The negative emotions “sad”, “scared” and “disgusted” were associated with the burger meals. Burgers involve ground or minced meat, which is visually and structurally completely different from a whole-muscle cut like a schnitzel. Research shows that ground meat categories, such as hamburger patties, are structurally associated with a higher perceived risk of microbial contamination, undercooking or foodborne illness. Encountering a burger format can unconsciously trigger avoidance behaviour specifically evoking disgust and “scared” (fear) regarding what is mixed into the meat or its degree of doneness [58].

### 4.6. The Role of Demographics in the Sensory Acceptance of Pork Meals

Socio-demographic characteristics including gender, age, education level and income significantly influenced (*p* < 0.001) consumer sensory acceptance of pork meals, according to the results in Table 3. This effect was primarily driven by whether bacon was present or absent in the meal. The gender differences align with established consumer behavioural patterns where men typically exhibit a stronger preference for processed meats and fat-associated savoury flavours (umami). Bacon acts as a flavour and aroma enhancer due to its concentrated fat content and the volatile chemical profiles released during cooking, drawing male interest via visual and olfactory cues [34]. Conversely, female consumers generally exhibit higher sensitivity to texture and meat quality, often favouring leaner options [59]. The preference of female respondents for burgers without bacon indicates an aversion to the greasy texture or overriding saltiness that bacon introduces, prioritizing the distinct textural and intrinsic flavour qualities of the core pork patty itself [59,60].

Younger consumers approach food choices through a dual lens of digital visual appeal and core sensory execution [61]. Researchers emphasize that visual presentation directly influences anticipated flavour and the overall enjoyment of the eating experience [62]. Older consumers focus primarily on visual cues, preferring rib burgers without bacon due to health-conscious habits [63].

### 4.7. Relevance of Putting Pork on the Menu

Convenience pork meat products and especially naturally wood-smoked products such as bacon often fall into the processed foods category; however, these products contain a good nutritional profile, especially the protein content having an average protein content of 16 g per 100 g serving. Of course, naturally wood-smoked products should still be consumed in moderation, as the World Health Organization has classified processed meats such as bacon, ham, sausages and salami as carcinogenic to humans (Group 1) [64]. Therefore, for general good health, it is recommended to consume bacon in moderation.

In the South African context, pork markets serve as pivotal contributors to food, nutrition, income, and employment across both formal and informal sectors. Numerous factors shape pork choice trends in South Africa, encompassing per capita income, religion population growth, urbanization, dietary diversity, limited availability of other meats and perceived quality of pig meat [65]. Consequently, the complete elimination of processed foods from the diet is projected to yield detrimental effects on public health, potentially diminishing the intake of essential food groups that provide vital nutrition and protective barriers against diet-related chronic diseases. A uniform recommendation to avoid all processed foods is fundamentally impractical. Such an approach lacks the necessary nuance to objectively assess the substantial nutritional contributions that various fortified, preserved, and convenient food products make to daily dietary requirements [66]. Therefore, removing all processed foods from the diet is likely to compromise population health, as it could reduce the intake of nutritionally adequate and disease-protective food groups. Furthermore, broadly discouraging all processed items lacks the nuance required to evaluate their substantial contributions to daily dietary intake [66].

This research study showed that adding bacon to pork meals can improve the sensory appeal of pork items, increasing their level of competition against other popular protein sources like beef or chicken. This kind of insight is vital for growing the local pork market. As perceptions of pork slowly shift in South Africa, understanding what drives consumer behaviour is essential. This study highlighted how enhancing the sensory experience of pork can help pork products to compete better in the protein market.

From an international perspective, this study advances sensory science by demonstrating how the sensory properties of naturally wood-smoked bacon and emotional triggers drive consumer choice. Concurrently, it offers the global food industry proven, consumer-driven insights to elevate the perceived value and market competitiveness of pork products on restaurant and fast-food menus. For policymakers and agricultural bodies, these findings offer a strategic pathway to design targeted local food campaigns that support domestic livestock sectors against global imports by leveraging regional culinary preferences. Finally, for consumers, the research translates into high-quality, emotionally satisfying dining experiences that align with a growing international demand for locally produced protein options. Food manufacturers can translate these findings into action by engineering premium, rindless streaky bacon with a higher meat-to-fat ratio, a light smoky aroma, a meaty flavour and a juicy yet crispy texture profile tailored for high-margin menu items.

### 4.8. Recommendations and Limitations

Some limitations for this study are that using bacon and convenience pork products from one retailer must be noted and considered when interpreting the results. It is recommended that more than one retailer of pork meat products should be used as the ingredients and processing may differ, which may affect the sensory aspects. Another limitation of this study is its restricted generalizability, as the findings may not represent the broader South African population or its diverse demographic groups. Consequently, future research should utilize a larger, more representative sample to enhance external validity. Furthermore, from a data analysis perspective, it is recommended to use a larger sample size to uncover the possibility of underlying associations between the sensory and emotional attributes of pork meat products.

While this study provides valuable insights into the sensory and emotional drivers influencing local pork selection, another limitation is its exclusive focus on sensory and affective measures, which overlook the confounding effects of consumer health literacy and nutritional awareness regarding pork’s sodium and saturated fat content. Furthermore, the methodology did not account for socio-religious constraints such as halal or kosher dietary laws that strictly prohibit pork consumption.

## 5. Conclusions

This study demonstrates that the inclusion of naturally wood-smoked bacon significantly enhances the sensory acceptability and emotional appeal of pork meals, highlighting its potential to drive local pork choices. Phase I identified rindless streaky bacon as the most accepted cut, characterized by a golden appearance, meaty and lightly smoky aroma, salty flavour and a juicy yet crispy texture. Across all evaluated cuts, the dominant emotional response elicited was happiness.

Phase II further established that adding streaky bacon to pork meals, most notably schnitzel and rib burgers, greatly increase positive consumer emotions, including “happiness”, “calmness” and “interest”, while altering the overall sensory profile of the dishes. Furthermore, the significant associations found between demographic variables (gender, age, education, and income) and meal acceptance emphasize the influence of inter-individual differences and oral processing on consumer preferences.

Ultimately, these findings suggest that the strategic incorporation of naturally wood-smoked pork cuts into restaurant and fast-food menus can successfully shift consumer perceptions, elevate the perceived value of pork, and foster positive market engagement. For pork manufacturers and culinary product developers, prioritizing the precise sensory qualities of bacon identified in this study offers a viable, consumer-directed pathway to improve the competitiveness of pork products within the broader protein market.

## Figures and Tables

**Figure 1:**
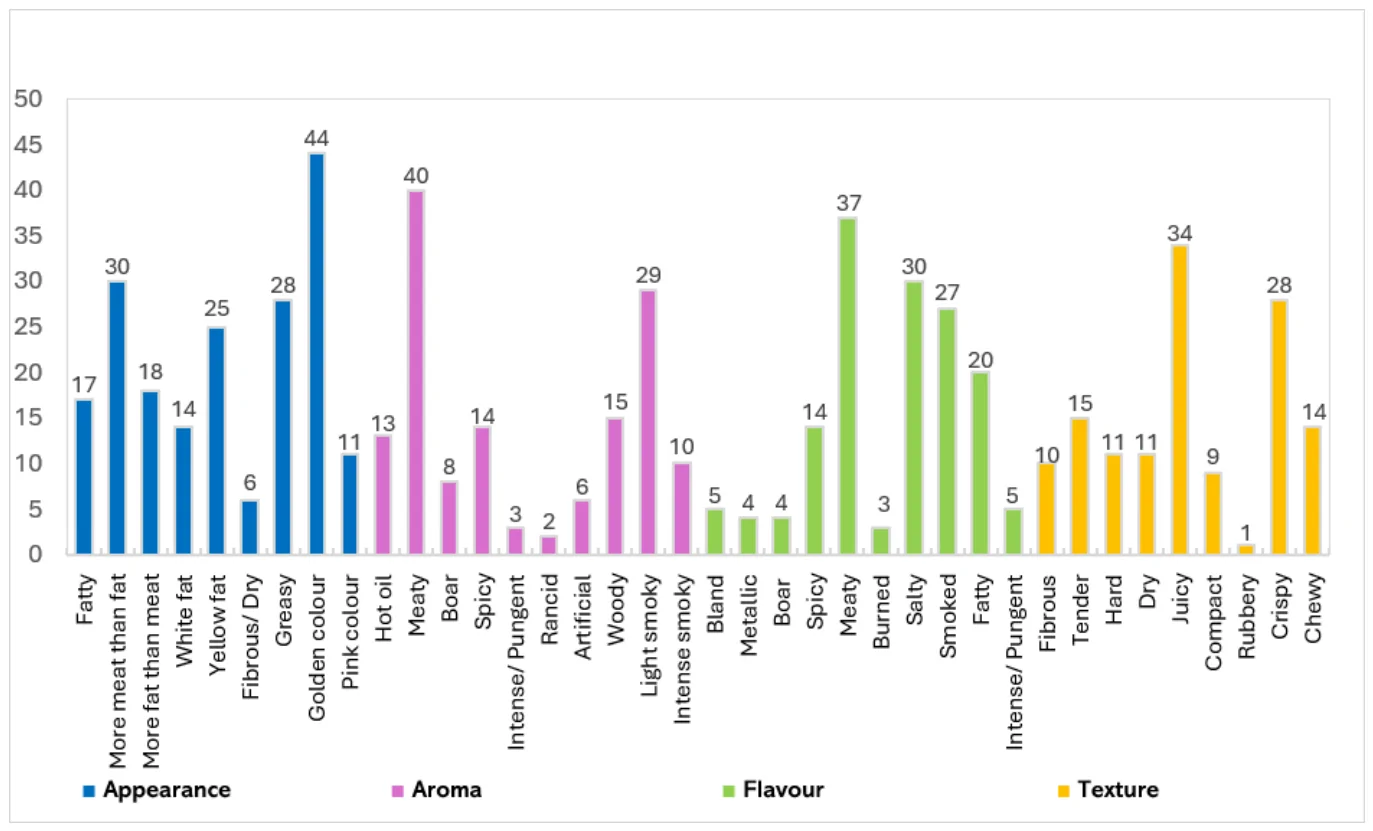
Frequency of sensory attributes (CATA) of rindless streaky bacon.

**Figure 2:**
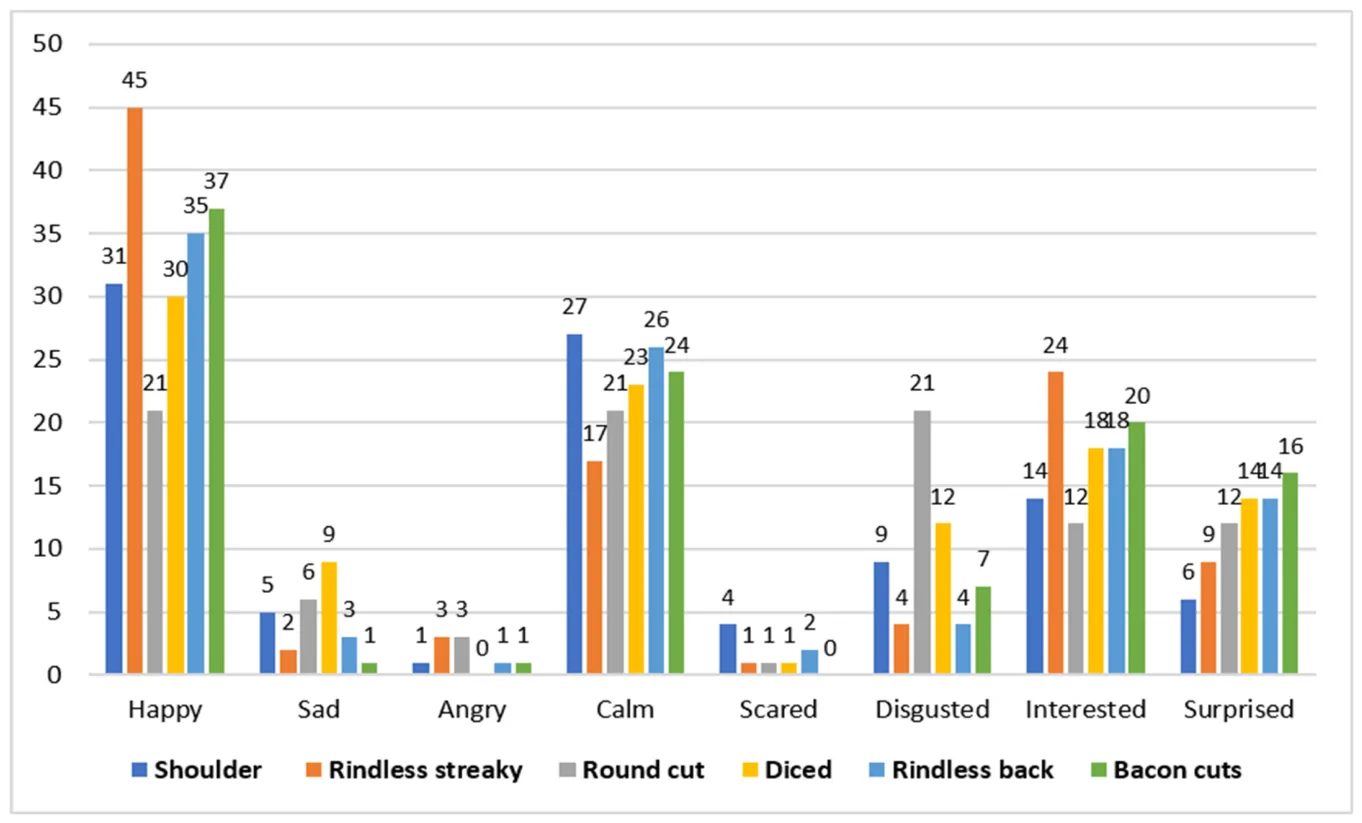
Frequency of emotions (CATA) for each naturally wood-smoked bacon cut.

**Figure 3:**
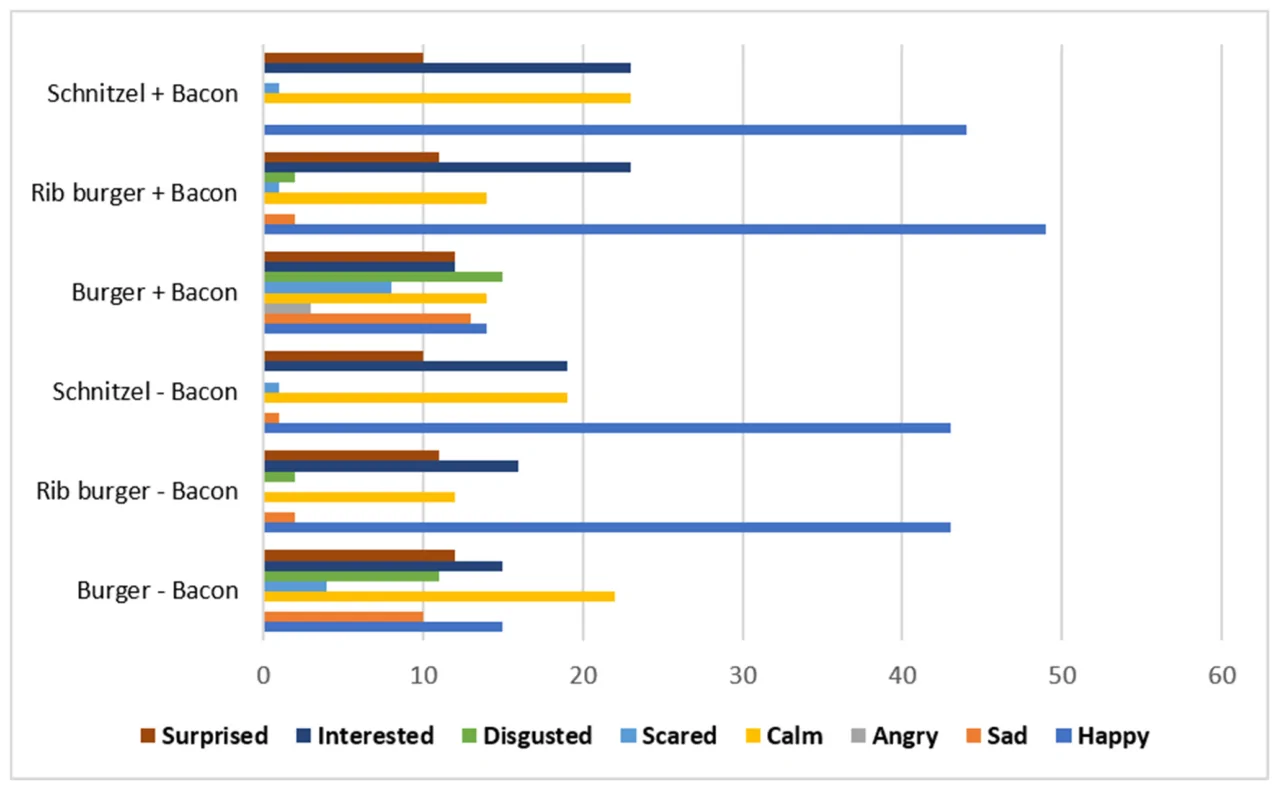
Frequency of emotions (CATA) for pork meals with and without bacon.

**Table 1:** Consumers’ (*n* = 76) sensory acceptability of naturally wood-smoked bacon.

	Shoulder	Rindless Streaky	Round Cut	Diced	Rindless Back	Bacon Cuts
Appearance	3.46 ± 1.87 ^ab^	2.57 ± 1.54 ^a^	4.03 ± 2.00 ^b^	3.87 ± 1.87 ^b^	3.04 ± 1.63 ^ab^	3.30 ± 1.64 ^ab^
Aroma	3.67 ± 1.96 ^b^	2.53 ± 1.28 ^a^	3.91 ± 1.99 ^b^	3.12 ± 1.79 ^ab^	3.25 ± 1.72 ^ab^	3.63 ± 2.10 ^b^
Flavour	3.68 ± 2.07 ^a^	2.47 ± 1.65 ^b^	4.13 ± 2.36 ^b^	3.37 ± 2.13 ^ab^	2.74 ± 1.70 ^ab^	3.00 ± 1.72 ^ab^
Texture	3.79 ± 2.07 ^ac^	2.68 ± 1.68 ^b^	4.41 ± 2.30 ^a^	3.88 ± 2.31 ^ac^	2.75 ± 1.71 ^bc^	3.13 ± 1.98 ^bc^

Note: Scale—1 = Like extremely; 2 = Like very much; 3 = Like moderately; 4 = Like slightly; 5 = Neither like nor dislike; 6 = Dislike slightly; 7 = Dislike moderately; 8 = Dislike very much; 9 = Dislike extremely; means with different superscripts in the same row differ significantly (*p* < 0.001).

**Table 2:** Consumers’ (*n* = 63) sensory acceptability of pork meals with and without streaky bacon.

		Without Bacon			With Bacon	
	Burger	Rib Burger	Schnitzel	Burger	Rib Burger	Schnitzel
Appearance	4.25 ± 1.73 ^a^*	2.16 ± 1.26 ^bc^	2.25 ± 1.14 ^b^	3.57 ± 1.79 ^a^*	2.24 ± 1.15 ^b^	1.94 ± 1.16 ^bc^
Aroma	4.06 ± 1.79 ^a^	2.54 ± 1.51 ^b^	2.37 ± 1.02 ^bc^	3.70 ± 1.79 ^a^	2.17 ± 1.29 ^b^	2.17 ± 1.19 ^bc^
Flavour	3.67 ± 1.95 ^a^	2.00 ± 1.41 ^b^	1.94 ± 0.97 ^bc^	4.21 ± 2.10 ^a^	2.25 ± 1.63 ^b^	1.89 ± 0.94 ^bc^
Texture	4.02 ± 2.03 ^a^	2.19 ± 1.42 ^b^	2.11 ± 1.19 ^bc^	4.44 ± 2.05 ^a^	2.05 ± 1.20 ^bc^	2.21 ± 1.14 ^b^

Note: Scale—1 = Like extremely; 2 = Like very much; 3 = Like moderately; 4 = Like slightly; 5 = Neither like nor dislike; 6 = Dislike slightly; 7 = Dislike moderately; 8 = Dislike very much; 9 = Dislike extremely; means with different superscripts in the same row differ significantly (*p* < 0.001); * Means differ significantly (*p* < 0.05) between pork meals.

**Table 3:** Statistical associations between demographics (*n* = 63) and the acceptance of pork meals without and with bacon.

Demographic	Appearance		Aroma		Flavour		Texture	
Gender Male Female	Burger − B	4.04 ± 1.51 *	RB − B	2.36 ± 1.50 *				
	Burger + B	3.24 ± 1.59 *	RB + B	1.80 ± 0.91 *				
					Burger − B	3.71 ± 1.94 *	Burger − B	4.05 ± 2.12 *
					Burger + B	4.45 + 2.19 *	Burger + B	4.66 + 2.20 *
Age 18–44 45–65+	Burger − B	4.4 ± 1.69 *			Burger − B	3.45 ± 1.78 *	Burger − B	3.85 ± 1.99 *
	Burger + B	3.5 ± 1.75 *			Burger + B	4.13 ± 2.08 *	Burger + B	4.5 ± 2.08 *
	RB − B	1.96 ± 0.83 *						
	RB + B	2.57 ± 0.95 *						
Highest level of education Grade 12 and below	Schnitzel − B	2.45 ± 1.21 *	Burger − B	4.55 ± 2.11 *	Burger − B	3.28 ± 1.85 *		
	Schnitzel + B	1.86 ± 1.33 *	Burger + B	3.62 ± 1.90 *	Burger + B	3.90 ± 2.04 *		
	Burger − B	4.59 ± 1.82 *	RB − B	2.72 ± 1.36 *				
	Burger + B	3.24 ± 1.70 *	RB + B	2.07 ± 1.51 *				
Gross Monthly Income (ZAR) 0–27,000	Burger − B	4.47 ± 1.73 *	RB − B	2.47 ± 1.50 *	Burger − B	3.53 ± 1.86 *	Burger − B	4.00 ± 2.07 *
	Burger + B	3.62 ± 1.83 *	RB + B	1.97 ± 1.55 *	Burger + B	4.35 ± 2.10 *	Burger + B	4.76 ± 2.06 *
			Burger − B	3.91 ± 1.31 *				
			Burger + B	3.41 ± 1.68 *				
27,001–100,000+			Burger – B Burger + B	3.91 ± 1.31 * 3.41 ± 1.68 *				

Note: B = Bacon; RB = Rib Burger; ZAR = South African Rand; Scale—1 = Like extremely; 2 = Like very much; 3 = Like moderately; 4 = Like slightly; 5 = Neither like nor dislike; 6 = Dislike slightly; 7 = Dislike moderately; 8 = Dislike very much; 9 = Dislike extremely; * Significance level = *p* < 0.001.

## Data Availability

The original contributions presented in the study are included in this article; further inquiries can be directed to the corresponding author.
